# The effect of metritis on luteal function in dairy cows

**DOI:** 10.1186/1746-6148-9-244

**Published:** 2013-12-04

**Authors:** Klaas Strüve, Kathrin Herzog, Fumie Magata, Marion Piechotta, Koumei Shirasuna, Akio Miyamoto, Heinrich Bollwein

**Affiliations:** 1 Clinic for Cattle, University of Veterinary Medicine Hannover, Bischofsholer Damm 15, D-30173, Hannover, Germany; 2 Graduate School of Animal and Food Hygiene, Obihiro University of Agriculture and Veterinary Medicine, Obihiro, Japan; 3 Clinic of Reproductive Medicine, University of Zurich, Zurich, Switzerland

**Keywords:** Metritis, Luteal size, Progesterone, Persistent corpus luteum, Gene expression, Bovine

## Abstract

**Background:**

Disturbed uterine involution impairs ovarian function in the first weeks after calving. This study analyzed the long-term effect of metritis on luteal function of 47 lactating Holstein-Friesian cows during the first four postpartum estrous cycles. Cows with abnormal uterine enlargement and malodorous lochia were classified as having metritis (group M, n = 18), and all others were considered healthy (group H, n = 29). Luteal size was measured once between days 9 and 13 of the first (group H, n = 11; group M, n = 12), second (group H, n = 23; group M, n = 18) and fourth (group H, n = 11; group M, n = 7) postpartum luteal phases. Serum progesterone concentration was measured at the same time. Sixteen cows (group H, n = 9; group M, n = 7) underwent transvaginal luteal biopsy for gene expression analysis of steroidogenic regulatory proteins during the second and fourth cycles. Cows with persistence of the corpus luteum (CL) underwent determination of luteal size, luteal biopsy and serum progesterone measurement once between days 29 and 33, followed by prostaglandin treatment to induce luteolysis. The same procedures were repeated once between days 9 and 13 of the induced cycle.

**Results:**

The cows in group M had smaller first-cycle CLs than the cows in group H (p = 0.04), but progesterone concentrations did not differ between groups. Luteal size, progesterone concentration and gene expression did not differ between the two groups during the second and fourth cycles. Compared with healthy cows (10%), there was a trend (p = 0.07) toward a higher prevalence of persistent CLs in cows with metritis (33%). Persistent CLs were limited to the first cycle. Persistent CLs and the induced cyclic CLs did not differ with regard to the variables investigated.

**Conclusions:**

An effect of metritis on luteal activity was apparent in the first postpartum estrous cycle. However, after the first postpartum cycle, no differences occurred in analyzed parameters between metritis and control cows. Therefore, a metritis is able to impair luteal activity transiently, but does not seem to have a long-term effect on luteal function.

## Background

Disturbed uterine involution has a significant adverse effect on reproductive performance in dairy cows [1-3]. Compared with healthy cows, days open was increased by 36 days in cows with endometritis [4], and twice as many affected cows were culled because of poor fertility [5]. Interestingly, for many years, there has never been a consistent definition of endometritis and metritis [5]. Sheldon et al. [6] characterized metritis by the presence of an enlarged uterus and a watery reddish-brown fluid to viscous off-white purulent uterine discharge, which often has a fetid odor. Whereas metritis occurs within 21 days, clinical endometritis is defined in cattle as the presence of a purulent uterine discharge detectable in the vagina 21 days or more post partum [6].

Uterine infections negatively affect ovarian activity. In cows with severe bacterial uterine contamination, the first postpartum dominant follicle was smaller and secreted less estradiol compared with healthy cows [7,8]. These cows also had smaller CLs and lower plasma progesterone (P4) concentrations than healthy cows [8].

Several authors have proven an association between disturbed uterine involution and the occurrence of persistent CLs in dairy cows [9-11]. Furthermore, the persistent CL has been determined as one of the most frequent abnormal ovarian activity in dairy cows [9,10]. The impact of persistent CLs on bovine fertility is largely unknown. While Taylor et al. [10] found no difference in reproductive competence between cows with persistence of the CL and normal cyclic cows, another study [12] showed that persistent CLs resulted in a higher level of late embryonic to early fetal mortality. It is generally accepted that if a CL fails to lyse and maintains its function beyond the physiological length of the estrous cycle, it is defined as a persistent CL. However, a uniform length of the luteal phase for diagnosing a persistent CL does not exist as yet in literature. Based on serial P4 measurements in blood or milk, a prevalence of 11 to 35% for persistent CLs has been calculated [9,10,12-16]. Although the pathogenesis of the formation of a persistent CL is not clearly understood, some studies give evidence that the luteotropic prostaglandin (PG) E might be involved [17,18]. Intrauterine infusion of PGE prolongs the luteal phase in cattle [19,20], and induction of luteolysis was blocked when PGF_2α_ and PGE were given to sheep simultaneously [21].

Luteal function can be evaluated by determining the gene expression of three specific proteins, which basically regulate the transformation of cholesterol to progesterone [22]. The steroidogenic acute regulatory protein (StAR) transports cholesterol from the outer to the inner mitochondrial membrane [23]. There, the cytochrome P450 catalyzes the conversion of cholesterol to pregnenolone, which is transformed to progesterone by the 3β-hydroxysteroid-dehydrogenase (3β-HSD; [22]). In the ovine CL, the average concentration of mRNA for cytochrome P450 increased from day 3 to day 9 (day 0 = estrus) and decreased significantly by day 15 [24]. The induction of luteolysis with the consecutive decrease of the plasma progesterone caused a significant reduction of the bovine gene expression of StAR and 3β-HSD [25].

The primary goal of the present study was to examine whether the effect of metritis on luteal function is limited to the first postpartum cycle [8] or whether subsequent cycles are also affected. A secondary aim was to examine whether a metritis induces the occurrence of a persistent CL. Furthermore, normal cyclic and persistent CLs were compared with regard to size, P4 secretion and various gene expressions.

## Methods

### Cows

Forty-seven primiparous and pluriparous lactating Holstein-Friesian cows from a research farm of the University of Veterinary Medicine Hannover, Germany, were used in the study. Cows were milked twice a day. Cows were 38 ± 12 [median ± MAD] months old, weighed 616 ± 66 kg and had a 305-day milk yield of 9085 ± 1657 kg. The cows were fed a total mixed ration, and concentrate was supplemented according to production. The study was approved by the independent ethics committee of the Lower Saxony Federal State Office for Consumer Protection and Food Safety, Oldenburg, Germany (research permit number 33.9 – 42502 – 04-09/1782).

### Group allocation

**Figure 1:**
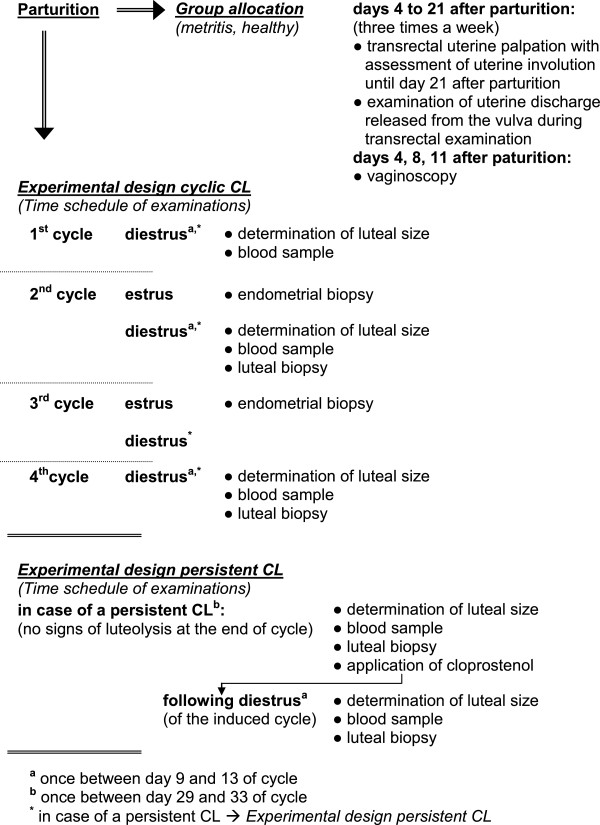
Time schedule of examinations.

Based on the results of the clinical examination of the reproductive tract, the cows were classified as healthy (group H) or as having metritis (group M; Figure 1). Uterine size was assessed subjectively according to a defined score system [26]. Transrectal palpation was carried out on days 4, 8 and 11 (±1 day) post partum and then three times a week until day 21. On day 21 post partum, cows with a uterus which could be gathered up with the hand, and horns of the uterus were the thickness of three or four fingers [26] or larger, were assumed to have a delayed uterine involution. When uterine discharge was released from the vulva during transrectal examination, it was noted, and its odor assessed. Furthermore, vaginoscopy was carried out using a tube speculum on days 4, 8 and 11, and the odor of the lochia was assessed. All examinations were carried out by the same person.

Based on Sheldon et al. [6], cows with a uterine enlargement and discharge having an offensive odor at any point of the examination were assigned to group M, and all other cows to group H. Metritis-cows which had fever (body temperature > 39.5°C) were treated daily for 3 days with ceftiofur (2.2 mg/kg, im, Excenel RTU®, Pfizer Animal Health, Germany) and on the day of diagnosis with a single dose of flunixin meglumine (2.2 mg/kg, iv, Finadyne®, MSD Intervet, Germany).

### Observation of cyclicity

Cows were monitored by ultrasonography three times a week (Monday, Wednesday, Friday) to detect the first ovulation starting on day 11 post partum. Ovulation was diagnosed when a dominant follicle, which had been seen at the previous examination, was no longer visible. A CL with a diameter of more than 7 mm was deemed to be the result of ovulation two days previously (day of ovulation = day 1), and the cycle stage was designated as day 3. When the dominant follicle was no longer present, and a CL was not visible or was smaller than 7 mm, the previous day was considered the day of ovulation. On days 5 ± 1 and 11 ± 2 of cycle, the presence of a CL was verified sonographically. The following three ovulations were diagnosed accordingly. To determine the occurrence of the second ovulation, monitoring (three times per week) started on day 14, and for detection of the third and fourth postpartum ovulations, monitoring (three times per week) started on day 18 of the previous cycle.

### Examinations of cyclic corpora lutea

Luteal size was measured sonographically once between days 9 and 13 of the first, second and fourth cycles, and blood samples were collected at the same time for measuring the serum P4 (Figure 1). In the first, second and fourth cycles, the CL of 23 (group H, n = 11; group M, n = 12), 41 (group H, n = 23; group M, n = 18) and 18 cows (group H, n = 11; group M, n = 7) was measured, respectively. At the beginning of the study, cyclic CLs of the second and fourth postpartum cycles were analyzed to examine the long-term effect of metritis on luteal activity. An additional number of cows was investigated during the first and second postpartum estrous cycles to evaluate the short-term effect of metritis on CL. All cows were grouped together to simplify the presentation of results. For that reason, the number of individuals in the second cycle included most of the animals. During the second and fourth cycles, 16 cows (group H, n = 9; group M, n = 7) underwent transvaginal biopsy of the CL once between days 9 and 13. Luteal biopsies were taken as described by Onnen-Lübben et al. [27] and Herzog et al. [28]. Cows which did not ovulate within the first 42 days post partum were excluded from analysis regarding cyclic CLs.

### Examinations of persistent corpora lutea

Cows with a CL with no sign of luteolysis at the end of a cycle underwent sonographic luteal examination and transvaginal luteal biopsy once between days 29 and 33 of the estrous cycle as well as blood collection. The existence of a persistent CL was confirmed by a serum P4 concentration of ≥ 1.0 ng/mL. After sonographic examination of the persistent CL, the cows were treated with cloprostenol (0.5 mg, im, Estrumate®, Intervet, Unterschleißheim, Germany) to induce luteolysis, and the subsequent ovulation was diagnosed as described for spontaneous ovulations. In the following induced cycle, these cows underwent another luteal biopsy as well as sonographic luteal examination and blood collection once between days 9 and 13 of the induced cycle (Figure 1).

### Endometrial biopsy and fertility

To verify whether the clinically based diagnosis of metritis leads to endometritis, transvaginal endometrial biopsies were taken during the second and third postpartum estrus (Figure 1). Biopsies were taken when a dominant follicle (Ø ≥ 10 mm) and only a small CL (Ø < 10 mm) were apparent during transrectal, sonographic examination. Artificial insemination was started at the fourth postpartum estrus. Only cows observed in standing heat were bred. Two pregnancy examinations were carried out using sonography on days 29 ± 1 and 40 ± 3 after breeding. An embryonic heart beat was used to confirm pregnancy. Days to first service and days open as well as insemination (total number of inseminations in all cows per number of pregnant cows) and pregnancy indices (number of inseminations per achieved pregnancy) were calculated for cows in the two groups. Cows not ovulating within the first 42 days post partum were excluded from analysis regarding endometrial biopsies and fertility.

### Ultrasonography

A portable ultrasound machine (HS-101 V, Honda Electronics CO., Tokio, Japan) with a 5-MHz linear-array transducer was used to monitor cows for ovulation, and another machine (LOGIQ Book XP, General Electric Medical System, Solingen, Germany), equipped with a 10-MHz linear-array transducer, was used to measure luteal size. According to Lüttgenau et al. [29], a CL was assumed to have the shape of a prolate spheroid. For each detectable CL, maximum longitudinal and cross-sectional images were frozen and recorded three times. The maximum height and width of the cross-sectional area of the CL were measured (PixelFlux Version 1.0, Chameleon Software, Leipzig, Germany) and taken as the major and minor diameters of the spheroid, respectively. The volume of the CL was calculated as follows:

$$\mathrm{volume}\;=\;4/3\;\times \;\pi \;\times \;a/2\;\times \;{\left(b/2\right)}^{2}$$

with *a* = major diameter (rotational axis) and *b* = minor diameter (transverse axis). If a CL had a cavity, the volume of the cavity was determined accordingly and subtracted from the previously calculated luteal volume. The difference between the total volume of the CL and the volume of its cavity was defined as the volume of luteal tissue. In double ovulations, the volumes were added, based on a previous study showing that the weight of a CL resulting from a single ovulation does not differ from the combined luteal weight resulting from a double ovulation [30].

### Measurement of progesterone and prostaglandin E

Blood samples from a jugular vein were collected (serum- and EDTA-tubes, Sarstedt, Nümbrecht, Germany) and placed on ice. Serum and plasma were separated by centrifugation (3 000 × g, 15 min) and frozen at −20°C. Serum P4 concentration was determined using a commercial coat-a-count radioimmunoassay according to the manufacturer’s instructions (Progesterone Coat-a-Count, TKPG1, Siemens Medical Diagnostics, CA, USA). For PGE analysis, cows of both groups were sampled once during the first postpartum luteal phase (between days 9 and 13) and cows with a persistent CL were tested between days 29 and 33 after the first ovulation. As described by Herzog et al. [28], PGE plasma concentration was determined using a commercial PGE2 enzyme immunoassay (Prostaglandin E2 EIA Kit, Biotrend Chemikalien GmbH, Cologne, Germany).

### Luteal RNA extraction and cDNA production

Luteal biopsies were used to quantify the levels of mRNA for StAR, cytochrome P450 and 3β-HSD. These substances are important factors for luteal steroidogenesis [22,24,25,31]. As described by Shirasuna et al. [32], total RNA was extracted from luteal biopsy samples, and cDNA was synthesized.

### Real-time reverse transcription-polymerase chain reaction of luteal biopsy

Levels of mRNA for two housekeeping genes, glycerolaldehyde-3-phosphate-dehydrogenase (GAPDH) and β-actin, as well as for StAR, cytochrome P450 and 3β-HSD were quantified by real-time PCR with a LightCycler (Roche Diagnostics Co.) using a commercial kit (LightCycler FastStart DNA Master SYBR Green I: Roche Diagnostics Co.). Based on bovine sequences, primers were designed using Primer-3. Primers used for real-time PCR were as follows: StAR (GenBank: MN174189) forward 5-GTG GAT TTT GCC AAT CAC CT-3 and reverse 5-TTA TTG AAA ACG TGC CAC CA-3; cytochrome P450 (GenBank: K02130) forward 5-CTG CAA ATG GTC CCA CTT CT-3 and reverse 5-CAC CTG GTT GGG TCA AAC TT-3; 3β-HSD (GenBank: X17614) forward 5-TCC ACA CCA GCA CCA TAG AA-3 and reverse 5-AAG GTG CCA CCA TTT TTC AG-3; GAPDH (GenBank: NM001034034) forward 5-CTC TCA AGG GCA TTC TAG GC-3 and reverse 5-TGA CAA AGT GGT CGT TGA GG-3; β-actin (GenBank: K00622) forward 5-CCA AGG CCA ACC GTG AGA AAA T-3 and reverse 5-CCA CAT TCC GTG AGG ATC TTC A-3. The amplification program consisted of 15 min activation at 95°C followed by 40 cycles of PCR steps (15 sec denaturation at 94°C, 30 sec annealing at 58°C and 20 sec extension at 72°C). For the quantification of the target genes, a series of standards were constructed by amplifying a fragment of DNA (150 ~ 250 bp) containing the target sequence for real-time PCR. The PCR products were subjected to electrophoresis, and the target band cut out and purified using a DNA purification kit (SUPRECTM-01, TaKaRa Bio. Inc., Otsu, Japan). The quantification of mRNA expression was performed using Light Cycler Software (Version 3.5, Roche). Primer sets were tested in luteal tissue samples to confirm amplification of single bands. Amplified products were cloned and sequenced to confirm their identity before using primers for analyzing the samples. The values determined for the target genes were normalized against the housekeeping genes GAPDH and β-actin (∆C_q_). To avoid negative digits while allowing an estimation of a relative comparison between two genes, data were presented as means ± SD subtracted from the arbitrary value 20 (∆C_q_). Thus, a high ∆C_q_ proportionally resembled high transcript abundance [33].

### Procedure of endometrial biopsy

The biopsy instrument, designed by Kevorkian (Fa. Hauptner Herberholz GmbH & Co. KG, Solingen, Germany), was used to collect endometrial tissue samples from both uterine horns approximately 2 cm cranial to the bifurcation. The instrument was introduced into the uterus analogous to an AI pipette. Tissue samples were fixed in 10% neutral formalin, buffered according to Lillie and embedded in paraffin. Sections, 3 to 4 μm thick, were stained with hematoxylin and eosin and examined microscopically for signs of inflammation including the occurrence of lymphocytes, plasma cells, macrophages and neutrophils. Endometritis was defined as an inflammatory cell infiltration of the endometrium which exceeded the normal cellular infiltration associated with repair of the endometrium. Up to 20 neutrophils in the luminal epithelium and 15 mononuclear cells (lymphocytes, plasma cells, macrophages) in the stratum compactum per high-power (x400) field are considered normal.

### Statistical analysis

The program SigmaStat 2.03 (Systat Software GmbH, Erkrath, Germany) was used for statistical analysis. Continuous variables were analyzed for normal distribution using the Kolmogorov-Smirnov test. Normally-distributed data are given as mean ± standard deviation. Differences between independent and dependent samples were analyzed using a Student’s *t*-test and a paired *t*-test, respectively. Data not normally distributed are presented as the median and mean absolute deviation (MAD). Differences between paired and unpaired samples were analyzed using the Wilcoxon signed ranks test and Mann–Whitney rank sum test, respectively. Most variables had a normal distribution (mean ± SD), and only those which did not were reported as median ± MAD. Days open of groups H and M were plotted by Kaplan Meier curves, and curves were compared by the Mantel-Cox test. Categorical data, such as histopathological findings or occurrence of a persistent CL, were analyzed using a chi-square test or Fisher’s exact test. A p-value ≤ 0.05 was considered significant, and a value 0.05 < p < 0.10 was considered as a trend toward significance.

## Results

### Groups

Based on the examinations up to day 21 post partum, 29 cows were healthy (group H) and 18 had metritis (group M). Three metritis-cows had fever between days 5 and 10, and were treated using the mentioned protocol. The two groups did not differ with regard to the interval from calving to first ovulation (group H, n = 29, 19 ± 7 days [median ± MAD]; group M, n = 18, 16 ± 3 days [median ± MAD]; p = 0.22). Six of 29 (21%) in group H did not ovulate within 42 days of parturition and none (0%) in group M (p = 0.07). No difference was found in age (p = 0.83), weight (p = 0.37) and 305-day milk yield (p = 0.93) between cows of both groups.

### Cyclic corpora lutea

In the first cycle, the luteal volume in cows of group M was smaller than that in cows of group H (p = 0.04; Table 1). In the twelve cows of group M, the first-cycle CL was smaller compared to their second-cycle CL (5.8 ± 2.9 versus 7.9 ± 1.9 cm^3^; p = 0.05, data not shown).

**Table 1:** Luteal size and progesterone of cyclic corpora lutea

	**1st cycle**	**2nd cycle**	**4th cycle**
Luteal size			
Group H	8.67 ± 3.36^*^ (11)	8.19 ± 2.69 (23)	9.08 ± 2.49 (11)
Group M	5.81 ± 2.86^*^ (12)	7.91 ± 2.46 (18)	7.41 ± 2.78 (7)
Progesterone			
Group H	4.22 ± 1.44 (11)	5.08 ± 1.18 (23)	5.08 ± 2.14 (11)
Group M	3.75 ± 1.81 (12)	4.67 ± 1.59 (18)	4.47 ± 1.22 (7)

Luteal size (cm^3^; mean ± SD) and serum progesterone concentration (ng/mL; mean ± SD) in healthy cows (group H) and cows with metritis (group M) with a cyclic corpus luteum in the first, second and fourth cycles (number of cows).

^*^p < 0.05 within a column.

Between cows of groups H and M, there were no differences in P4 concentrations of all three analyzed cycles (p ≥ 0.35; Table 1). In the first, second and fourth postpartum cycles, the time of assessment of luteal size and P4 concentration did not differ between the two groups (p ≥ 0.34). Assessments were carried out on days 27 ± 6 (group H) and 28 ± 5 (group M) in the first cycle, on days 54 ± 13 (group H) and 56 ± 9 (group M) in the second cycle and on days 102 ± 11 (group H) and 101 ± 7 (group M) in the fourth cycle, respectively.

In healthy cows, there were differences in gene expression for cytochrome P450 and 3β-HSD between the second and fourth cycles (Table 2, both p = 0.02).

**Table 2:** Gene expression of cyclic corpora lutea

	**2nd cycle**	**4th cycle**
StAR		
Group H	1.11 ± 0.42 (9)	0.88 ± 0.29 (9)
Group M	0.98 ± 0.30 (7)	1.17 ± 0.39 (7)
Cytochrome P450		
Group H	1.04 ± 0.60^*^ (9)	1.46 ± 0.64^*^ (9)
Group M	1.21 ± 0.57 (7)	1.11 ± 0.55 (7)
3β-HSD		
Group H	1.37 ± 0.72^*^ (9)	0.68 ± 0.25^*^ (9)
Group M	1.27 ± 0.79 (7)	0.74 ± 0.51 (7)

mRNA expression (ΔCq; mean ± SD) in healthy cows (group H) and cows with metritis (group M) for steroidogenic acute regulatory proteins (StAR), cytochrome P450 and 3β-hydroxysteroid-dehydrogenase (3β-HSD) of luteal tissue during the second and fourth postpartum cycles (number of cows).

^*^p < 0.05 within a row.

### Persistent corpora lutea

Of the nine cows with a persistent CL, six belonged to group M and three to group H (6/18: 33% versus 3/29: 10%; p = 0.07). All persistent CLs occurred in the first luteal phase. The single treatment with prostaglandin induced luteolysis in all cases of a persistent CL. No difference was found between luteal sizes, P4 concentrations and gene expressions (StAR, cytochrome P450, 3β-HSD) of persistent CLs and the CLs which formed after induced luteolysis of the former (p ≥ 0.20). There was no difference between the PGE concentrations of cows with a persistent CL (blood sample taken after CL biopsy) and cows in both groups (H and M) with a normal cyclic CL (blood sample taken once between days 9 and 13) during the first postpartum cycle (3.0 ± 1.5 ng/mL versus 3.45 ± 1.54 ng/mL; p = 0.43).

### Endometrial biopsy and fertility

**Figure 2:**
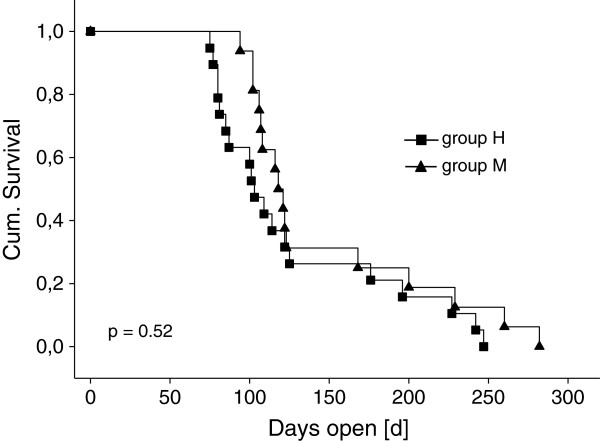
Kaplan Meier curves for days open [d] of healthy cows (■; group H; n = 22) and cows with metritis (▲; group M; n = 18).

In group M (n = 18), there was a trend toward more cows with histological signs of endometritis than in group H (n = 23) during the second estrus (67% versus 35%; p = 0.06). In the third estrus, there were significantly more cows showing signs of endometritis in group M compared to group H (56% versus 16%; p = 0.02). Two cows each from both groups were excluded from breeding after five unsuccessful inseminations. Days to first insemination (group H, 101 ± 18 days; group M 100 ± 17 days; p = 0.90) and days open (Figure 2; p = 0.52) did not differ between the two groups. Neither differences in insemination nor in pregnancy indices (p ≥ 0.25) were found between groups H (2.2 and 1.7) and M (2.9 and 2.3).

## Discussion

In cows with metritis, the CLs of the first postpartum estrous cycle were smaller than those of the second cycle, and they were also smaller than the first-cycle CLs of healthy cows. This was in agreement with observations made by Williams et al. [8] that the occurrence of large numbers of pathogenic bacteria in the uterine lumen is associated with smaller first postpartum CLs. Brinsfield and Hawk [34] demonstrated that intrauterine infusion of *E. coli* during the previous estrous period caused a reduction in the luteal weight in cows. The pathogenesis of luteal impairment through metritis remains unclear. Metritis causes increased PGF_2α_ plasma concentrations [35-37], which possibly disturb luteal development. Other inflammatory mediators, such as tumor necrosis factor-α which may be released during metritis, are cytotoxic to bovine luteal cells [38]. Furthermore, endotoxin inhibits the responsiveness of the pituitary to GnRH [39], which in turn could affect ovulation and luteal development.

In addition to smaller CLs in the first postpartum estrous cycle, bacterial contamination of the postpartum uterus has also been shown to be associated with lower plasma P4 concentrations [8]. This effect was not seen in the present study. Possible reasons for this discrepancy include the use of clinical criteria [6] rather than uterine culture results [8] for classification of the cows in the present study, as well as the time of P4 measurement. Whereas the lower plasma P4 concentrations in cows with high numbers of uterine pathogens were measured between days 21 and 26 post partum [8], many of our cows were sampled later because determination of P4 concentration of the first postpartum cycle was carried out on days 27 ± 6 (group H) and 28 ± 5 (group M) post partum, respectively. Uterine involution [40] and elimination of bacteria from the uterine lumen [41] progress continuously in the postpartum period, which was also the reason why cows which remained anovulatory beyond day 42 post partum were eliminated from luteal and hormonal analysis.

In addition to the evaluation of the uterine discharge, cows were classified as healthy or having a metritis based on the involution of the uterus. Although the transrectal palpation of the uterus is subjective [40], and thresholds for uterine enlargement do not exist in literature, it has been demonstrated that an infection of the uterus delays the postpartum involution of the uterus [11,42]. In the present study, the validity of the applied group allocation was confirmed by the histological examinations of endometrium biopsies because cows in group M had a higher prevalence of endometritis in the second and third postpartum estrus compared to cows in group H.

The results of gene expression of enzymes involved in progesterone synthesis were surprising due to differences between the second and fourth cycles in cows of group H (Table 2). Expression of cytochrome P450, which converts cholesterol to pregnenolone [43], was reduced and expression of 3β-HSD, which converts pregnenolone to progesterone [22], was increased in the second cycle compared with the fourth cycle. We have no clear explanation for this difference, but this could be a kind of complementary mechanism by which the CL maintains a stable amount of P4 synthesis and secretion. This idea is supported by the result that peripheral P4 concentration and expression of StAR did not differ significantly between the second and fourth cycles, in agreement with the regulatory function of the StAR protein on peripheral P4 concentration documented in pigs [23].

There was a trend toward an increased prevalence of persistent CLs in cows with metritis, in agreement with previous reports stating that disturbed uterine involution may be associated with this condition [9-11]. The prevalence of persistent CLs calculated in the present study was 19%, which was similarly reported elsewhere [9,10,12,15,16]. However, in contrast to our study, persistent CLs were not always limited to the first postpartum cycle [10,12,16]. In those studies, the presence of luteal tissue was monitored indirectly using serial P4 measurements in blood or milk, rather than directly depicting luteal tissue using sonography. It is therefore possible that in the cited studies at least some persistent CLs were mistaken for luteinized ovarian cysts, which can cause prolonged elevation of peripheral P4 concentration [44,45].

There were no differences between cyclic and persistent CLs with regard to size, peripheral P4 concentration and gene expression for StAR, cytochrome P450 and 3β-HSD, suggesting that the function of a persistent CL is not reduced compared with that of a cyclic CL. However, the cellular mechanisms involved in luteolytic disturbance remain elusive.

In addition to abnormal uterine PGF_2α_ secretion [37], inflammatory-mediated secretion of PGE, which is luteotropic [20], might be responsible for prolonged luteal function. However, cows with persistent CLs did not have elevated peripheral PGE concentration in our study. Therefore, a prolonged peripheral increase in PGE is not a likely mechanism of luteal persistence. Nonetheless, endometritis causes an increase in PGE concentration in uterine fluid of cows [37], and endometrial epithelial cells exposed to endotoxin undergo an endocrine switch from luteolytic PGF_2α_ to luteotropic PGE [46]. Increased PGE concentrations in uterine fluid cause luteal persistence in cows [18], suggesting that in cows with metritis this prostaglandin plays a local role in the pathogenesis of persistent CL.

Reduced feed intake and a negative energy balance are main factors leading to a delay of postpartum ovarian activity [47,48]. As metritis reduces the feed intake, an extended anovulatary postpartum period of cows with metritis compared to healthy cows could be expected [49]. Surprisingly, in the present study, there was a trend toward more healthy cows with the first ovulation after day 42 post partum than cows with metritis. This result, however, was relativized by the fact that healthy and metritis groups did not differ with regard to the interval from calving to first ovulation.

## Conclusions

In the present study, a negative effect of metritis on luteal activity was only verifiable in the first postpartum estrous cycle, at which time metritis was associated with a smaller luteal size and a trend toward increased prevalence of luteal persistence. An effect of metritis on the studied variables was not apparent after the first postpartum cycle. Therefore, a long-term effect on luteal function is not a likely cause of reduced fertility in dairy cows with metritis.

## Competing interests

The authors declare that they have no competing interests.

## Authors’ contributions

HB initiated the study, designed the study and participated in preparing and editing the manuscript. KST designed the study, performed the data collection, conducted the statistical modeling and drafted the manuscript. KH participated in the design of the study, supported the data collection and drafted the manuscript. AM, FM and KS analyzed luteal gene expressions and interpreted the results. MP performed the hormonal analyzes. All authors have read and approved the manuscript.
